# Prevention and management of postpartum hemorrhage and hypertensive disorders of pregnancy: results from a 31-country rapid assessment

**DOI:** 10.1186/s12884-026-09679-w

**Published:** 2026-07-20

**Authors:** Gaurav Sharma, Angeline Noriega, Aleefia Somji, Andre Blockett, Patricia Gomez, Leah Elliott, Blami Dao, Alma Virginia Camacho Hubner, Suzanne Stalls

**Affiliations:** 1 MOMENTUM and Jhpiego, The Johns Hopkins University Affiliate, Baltimore, MD USA; 2 Vancouver, WA USA; 3 Jhpiego Burkina Faso, Ouagadougou, Burkina Faso; 4 UNFPA Latin America and Caribbean Regional Office, Panama City, Panama

**Keywords:** Postpartum hemorrhage, Hypertensive disorders of pregnancy, Pre-eclampsia, Eclampsia, SDG 3, Maternal mortality, Neonatal mortality, Private sector

## Abstract

**Background:**

Postpartum hemorrhage (PPH) and Hypertensive Disorders of Pregnancy (HDP) remain leading causes of maternal death. This multi-country survey asked key informants to assess uptake of evidence-based practices; determine their country’s implementation of updated WHO global guidelines; and describe the private sector’s role in PPH and HDP management. To our knowledge, this is among the few multi-country assessments to examine national implementation of updated WHO recommendations for PPH and HDP alongside private-sector engagement across three major regions—sub-Saharan Africa, South and Southeast Asia, and Latin America and the Caribbean—providing a comparative view to guide advocacy and programme planning.

**Methods:**

From January to May 2022, 35 countries in sub-Saharan Africa, South and Southeast Asia, and Latin America and the Caribbean were invited to complete a survey (based on 2011 and 2012 surveys) on PPH and HDP management across public and private sectors on PPH and HDP management. Using purposive sampling, country-based focal persons assembled national expert teams from public and private sectors in health policy, education, procurement/distribution, logistics, and information systems to complete the survey. Teams produced consensus-based answers by reviewing nationally available data and policy documents. The Study Team developed composite scores for key quantitative indicators and thematically analyzed qualitative data.

**Results:**

Thirty-one countries completed the survey. Despite significant progress since 2012, several gaps remain. Oxytocin was reported as regularly available in public facilities by 77% of countries and in private facilities by 84% of countries. Magnesium sulfate was reported as available in public facilities by 58% of countries and in private facilities by 45% of countries. Interventions like umbilical vein injection of oxytocin for retained placenta (13% of countries) and heat-stable carbetocin for PPH prevention (45% of countries) were less integrated in national policy guidelines. Since 2011, midwifery scope of practice remained stagnant for manual removal of placenta for PPH, diagnosing severe pre-eclampsia/eclampsia, and giving the loading dose of MgSO4, but 60% of countries in 2022 reported that newer PPH treatments (such as tranexamic acid) were included. Quality assurance systems for procurement of essential drugs and data reporting into national information systems were deficient, particularly in the private sector. Conclusions: Recommendations include prioritizing integration of updated global guidelines; improving uptake through professional education, mentorship, and supervision; quality assurance; strengthening procurement and supply chains to improve access, availability, and quality of essential drugs and commodities; expanding midwives’ scope of practice; and improving private sector governance to enhance reporting of important MNH indicators.

**Supplementary Information:**

The online version contains supplementary material available at https://doi.org/10.1186/s12884-026-09679-w.

## Background

The current global maternal mortality ratio (MMR) target as set by the 2030 Sustainable Development Goal (SDG) 3 is fewer than 70 per 100 000 live births [1]. The latest maternal mortality estimates have suggested stagnation; at the current pace of progress, the World Health Organization (WHO) estimates that this target will not be reached [2]. While global MMR decreased from 339 per 100 000 live births in 2000 to 223 per 100 000 live births in 2020, these reductions have not been evenly distributed, with extremely high MMR in countries like South Sudan and Nigeria (1223 and 1047 per 100 000, respectively) compared to an MMR of 1 per 100 000 in Belarus. Sub-Saharan Africa and South and Southeast Asia carry a disproportionate burden of maternal mortality: 87% of global estimated maternal deaths in 2020 occurred in these regions. An estimated 800 women continue to die daily from complications of pregnancy and childbirth [2].

Addressing two of the leading causes of preventable maternal deaths—Postpartum Hemorrhage (PPH) and Hypertensive Disorders of Pregnancy (HDP)—is key to reducing maternal mortality worldwide. PPH affects millions of women every year and accounts for over 20% of all maternal deaths globally, while causing significant maternal morbidity [3]. HDP also causes severe morbidities, long-term disability, and deaths of pregnant women and their babies, accounting for approximately 14% of all maternal deaths worldwide and an estimated 10–25% of perinatal mortality—stillbirths and early neonatal mortality—with wide regional variation [4, 5].

Global recommendations and treatment guidelines to address PPH and HDP have been available for the past two decades. In 2011 and 2012, a key informant survey by the Maternal and Child Health Integrated Program (MCHIP) of the United States Agency for International Development (USAID) assessed the extent to which these global recommendations were integrated into national programs for PPH and HDP [6, 7]. Results from these surveys provided country and global-level snapshots of policies, practices, supplies, and activities to guide national and global program managers and policymakers in setting national priorities and managing their PPH and HDP programs [6, 7].

Since 2012, several critical changes have occurred in the global guidance for PPH and HDP, including new PPH treatments and more specific management guidelines for prevention, treatment and management of post-pregnancy risk factors for HDP [4, 8–16]. Additionally, the private sector has emerged as a significant provider of maternity services in many countries, although little is known about the extent to which evidence-based policies, commodities and practices are used in the private sector [17, 18]. To understand the current landscape of PPH and HDP programs globally, the USAID-funded MOMENTUM Country and Global Leadership and MOMENTUM Private Healthcare Delivery projects conducted an updated rapid assessment in 2022. The assessment was designed to take stock of the changes and best practices sustained in PPH and HDP management since 2012, identify how countries are implementing the updated WHO guidelines, understand the private sector’s role in national programs, and formulate recommendations for countries to effectively accelerate progress in reducing preventable maternal deaths from PPH and HDP. Practices assessed included WHO-recommended uterotonics for PPH prevention (oxytocin, heat-stable carbetocin), tranexamic acid for PPH treatment, and magnesium sulfate for HDP.

## Methods

### Design, setting and participants

From January to May 2022, MOMENTUM Country and Global Leadership and MOMENTUM Private Healthcare Delivery projects conducted a descriptive cross-sectional survey of countries in sub-Saharan Africa, South/Southeast Asia, and Latin America to assess country implementation of global best practices in PPH and HDP policies, commodities, and practices (See Table 1). Thirty-five countries were purposively chosen to participate based on (1) participation in a previous round of the survey; (2) USAID priority country designation; (3) UNFPA priority country designation (for Latin America and Caribbean (LAC) region countries); and (4) having a Jhpiego country presence. In each country, an expert group was established that typically had 10 to 20 participants and included representatives from the Ministry of Health; universities, professional education councils and professional associations spanning midwifery, nursing, and obstetrics-gynecology; private sector stakeholders from nongovernmental organizations; faith-based organizations; for-profit formal private sector entities; and United Nations agencies. Methodological details on the 2011 and 2012 surveys have been published previously [6, 7].

**Table 1:** Participating countries by region and year

Countries by Region	2011 (*n* = 31 countries)	2012 (*n* = 37 countries)	2022 (*n* = 31 countries)
	Total = 21	Total = 20	Total = 17
AFRICA			
1. Angola	x	x	
2. Burkina Faso			x
3. Côte d’Ivoire			x
4. Democratic Republic of the Congo	x	x	x
5. Ethiopia	x	x	x
6. Equatorial Guinea	x	x	
7. Ghana	x	x	x
8. Guinea	x	x	x
9. Kenya	x	x	x
10. Liberia	x	x	x
11. Madagascar	x	x	x
12. Malawi	x	x	x
13. Mali	x	x	x
14. Mozambique	x	x	x
15. Nigeria	x	x	x
16. Rwanda	x	x	
17. Sierra Leone			x
18. Senegal	x	x	
19. South Sudan	x	x	x
20. Tanzania	x	x	
21. Uganda	x	x	x
22. Zambia	x		x
23. Zanzibar	x	x	
24. Zimbabwe	x	x	
SOUTH/SOUTHEAST ASIA	Total = 5	Total = 10	Total = 6
25. Afghanistan	x	x	
26. Bangladesh	x	x	x
27. Burma (Myanmar)			x
28. Cambodia		x	
29. India	x	x	x
30. Indonesia	x	x	x
31. Nepal	x	x	x
32. Pakistan		x	x
33. Philippines		x	
34. Timor-Leste		x	
35. Yemen		x	
LATIN AMERICA/ CARIBBEAN	Total = 5	Total = 7	Total = 8
36. Bolivia	x	x	
37. Colombia			x
38. Dominican Republic			x
39. Ecuador		x	
40. El Salvador		x	x
41. Guatemala	x	x	x
42. Honduras	x	x	x
43. Nicaragua	x	x	
44. Paraguay	x	x	x
45. Peru			x
46. Uruguay			x

*Afghanistan, Pakistan, and Yemen from the WHO Eastern Mediterranean Region are included in S/SE Asia

*We used country classifications as previously used by Smith et al.(6). to ensure comparability over time and across regions

## Survey tool

The survey tool comprised 69 quantitative and qualitative questions, including 16 questions imported from the 2011 and 2012 surveys (and refined for clarity) to allow for temporal comparison (Additional File 1). New questions were added about private sector roles to reflect the growing role of the private sector in RMNCH [17]. The survey focused on seven themes: (1) essential drug availability; (2) national guidelines updated to recent global technical updates; (3) quality assurance and procurement policies at the national level; (4) midwifery scope of practice; (5) updated capacity building and training; (6) national reporting on selected maternal health indicators; and (7) bottleneck and scale-up opportunities. Survey questions assessed the reported inclusion of 23 current WHO guidelines for PPH and HDP drugs and practice guidelines into the responding country’s national guidelines [4, 8, 9, 11–16]. The survey tool was reviewed through an iterative process involving expert reviewers from the Postpartum Hemorrhage Community of Practice, USAID, Population Services International, and Jhpiego.

### Data collection

The survey was sent electronically to a national focal person in each country. The study team identified and confirmed the focal persons, who were typically affiliated with Jhpiego and who held various titles, from chief of party to consultant. UNFPA identified focal persons in all LAC countries. At the country level, a national focal person convened an expert group (average of 10 to 20 national experts) including members of maternal and newborn health technical working groups and other experts in leadership positions to discuss questions, reach consensus on survey responses, and complete one set of responses per country. Qualitative inputs were recorded as open-text responses to designated survey questions, documented by the expert group during the consensus discussions and confirmed with the national focal person. Surveys and informed consent were administered in English, French, Spanish, and/or Portuguese, as appropriate. A professional translation service was used for translations of tools from English to French and Portuguese; the Spanish translation was conducted by a bilingual technical advisor. Multiple attempts were made during data collection to communicate with and support countries and encourage their response. A separate health facility assessment was not conducted, and the data collected were obtained solely from expert consultations.

### Data analysis

The study team checked country surveys for completeness and clarity and contacted the country focal person regarding needed clarifications or missing data. Responses were analyzed across the public and private sectors, and temporal changes from 2011 to 2022 data were assessed where data were available. Quantitative data was analyzed using Microsoft Excel to calculate mean scores by region and themes. Composite scores were developed for all quantitative indicators. The composite score was calculated by highlighting key components of each theme, giving a score of one for a “yes” response per indicator, totaling the scores, dividing by the number of indicators per theme, and then converting this result to a percentage. Composite scores were expressed as the percentage of a theme’s component indicators met, such that a higher score reflects a more enabling national policy and programme environment for that theme; scores were not weighted by clinical importance and do not measure facility-level performance. Additional File 2 contains a complete list of the composite score indicators by theme. Key components per theme ranged from six to 40, but typically had 10–14 components. The analysis weighed all questions equally.

Qualitative responses were analyzed using a combined framework and inductive approach: coding in Microsoft Excel was organized around seven pre-defined thematic areas aligned with the survey domains, within which subthemes were developed inductively and retained where three or more similar responses occurred. Coding was conducted by a researcher and reviewed by the study team, with clarifications resolved with country focal persons, and qualitative findings were triangulated with quantitative results to enrich interpretation and elucidate bottlenecks and scale-up opportunities.

## Results

Of the 35 invited countries, 31 countries completed the survey (Table 1). Burundi, Niger, Rwanda, and Tanzania did not complete the survey.

Results are organized by core components identified in global recommendations as key elements of PPH and HDP national efforts. Survey data from 2022 are presented; where temporal comparative data are available, figures present data from 2011, 2012, and 2022. Temporal changes noted in the survey over the last decade can be interpreted as general trends for countries and regions.

A cross-cutting finding across themes was that quality assurance and routine reporting were consistently weaker in the private sector than in the public sector. Although most countries reported that private facilities follow national guidelines, private-sector performance lagged on controlled cold chain for oxytocin, systems to ensure correctly concentrated MgSO4, inclusion of recent PPH interventions in the midwifery scope of practice, and HMIS reporting—where only 45% and 52% of countries estimated that the private sector reports the PPH and HDP indicators, compared with 74% and 87% for the public sector. These gaps are detailed by theme below and recur as a central theme in the bottleneck and scale-up findings.

### Essential drug availability for PPH and HDP

**Fig. 1:**
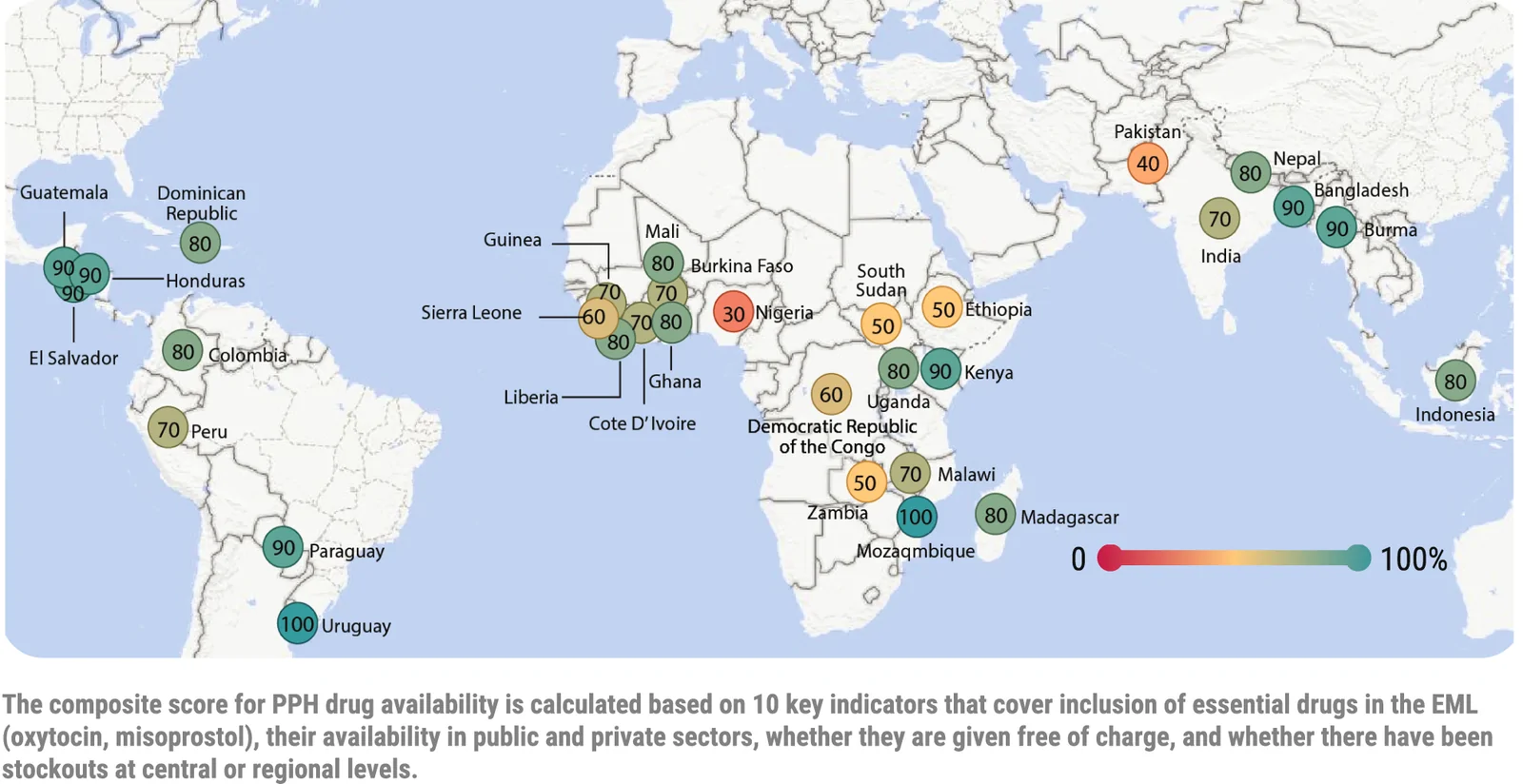
Overview of drug availability for PPH in 31 countries, 2022

**Fig. 2:**
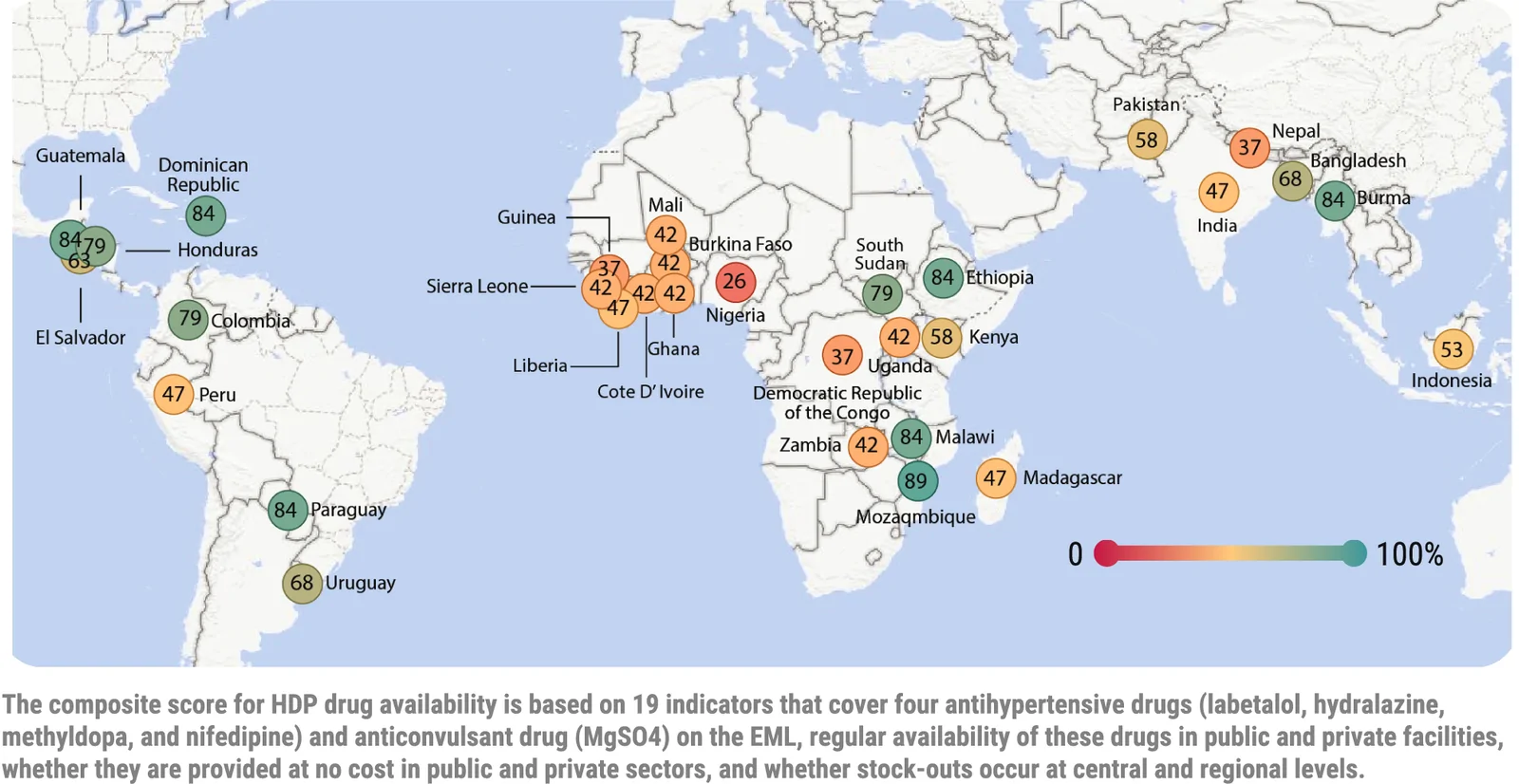
Overview of drug availability for HDP in 31 countries, 2022

Across the countries surveyed, 58% of countries scored 80% or higher for their composite score on uterotonic drug availability and 23% of countries scored 80% or higher for their composite score on drug availability for MgSO4 and anti-hypertensives for HDP (See Figs. 1 and 2 for drug availability composite scores by country). Drug availability varied globally, most notably for HDP drugs; countries in the LAC region reported greater HDP drug availability than West Africa and S/SE Asia. Regular availability was defined in the 2022 survey as available 80% or more of the time; in the 2011 and 2012 surveys, regular availability was not defined.

All countries reported having oxytocin, MgSO4, and at least one of the four anti-hypertensives recommended by the WHO for HDP (hydralazine, labetalol, methyldopa, and nifedipine). Nearly all countries reported having misoprostol on their Essential Medicine List (EML). For PPH, 35% reported heat-stable carbetocin and 71% of countries reported tranexamic acid as being on the EML for use in PPH management.

**Fig. 3:**
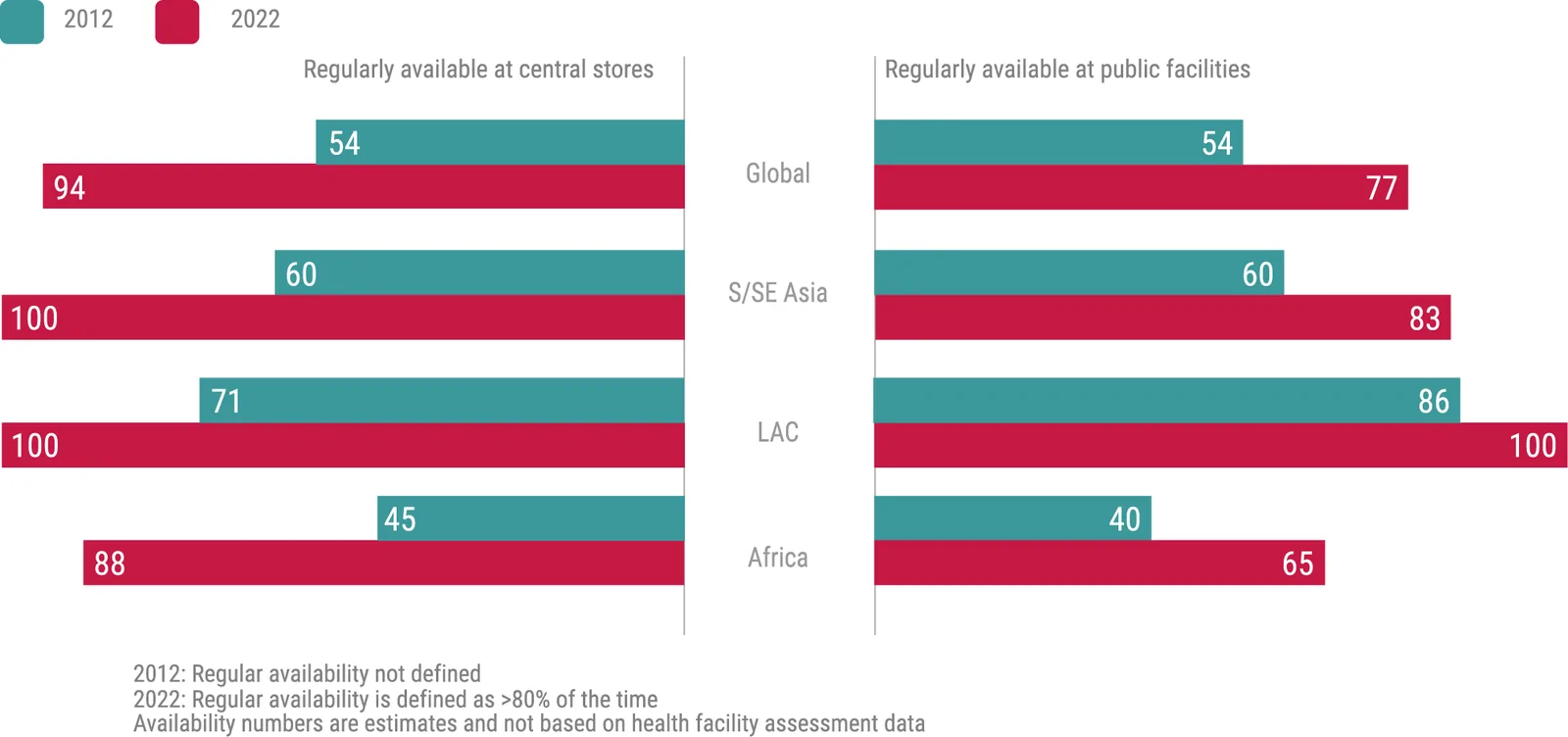
Percentage of countries estimating oxytocin availability at central stores and public facilities, 2012-2022

**Fig. 4:**
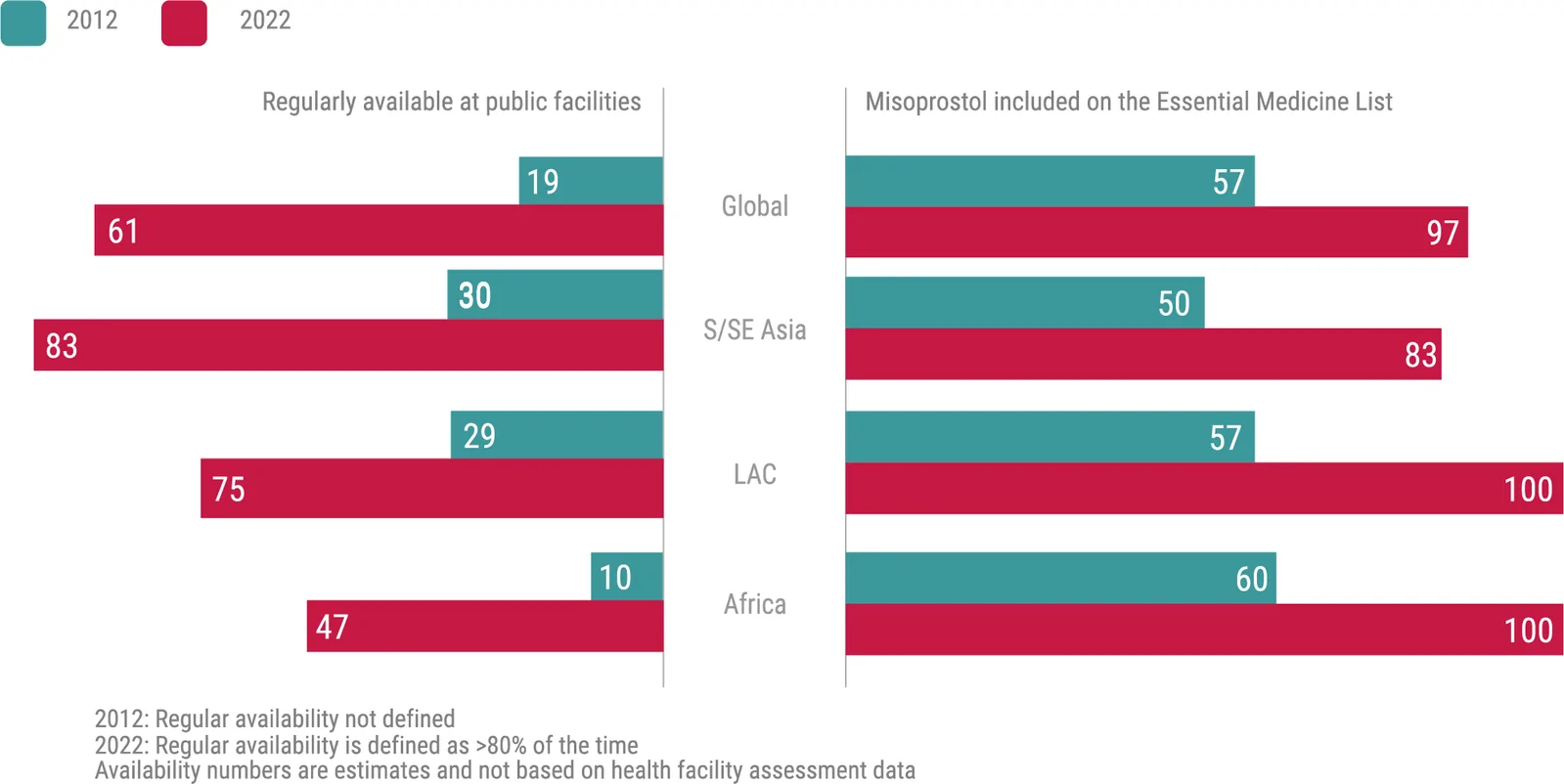
Percentage of countries estimating misoprostol availability at public facilities and inclusion on the Essential Medicine List, 2012-2022

Drug availability improved for uterotonics compared to anti-hypertensive or anti-seizure drugs from 2011 to 2022. In 2022, 94% of countries reported that oxytocin stockouts at central stores occurred rarely (i.e., once per year or less often), compared to 54% of countries in 2012. Regular availability of oxytocin at public facilities improved: 54% of countries reported regular oxytocin availability in 2012, compared to 77% in 2022. Misoprostol availability changed most significantly. From 2012 to 2022, countries reporting misoprostol on their EML nearly doubled from 57% in 2012 to 97% in 2022. In 2022, 61% of countries reported misoprostol as being regularly available at public facilities, compared to 19% in 2012. Figures 3 and 4 visualize these temporal changes in PPH drug availability by region.

**Fig. 5:**
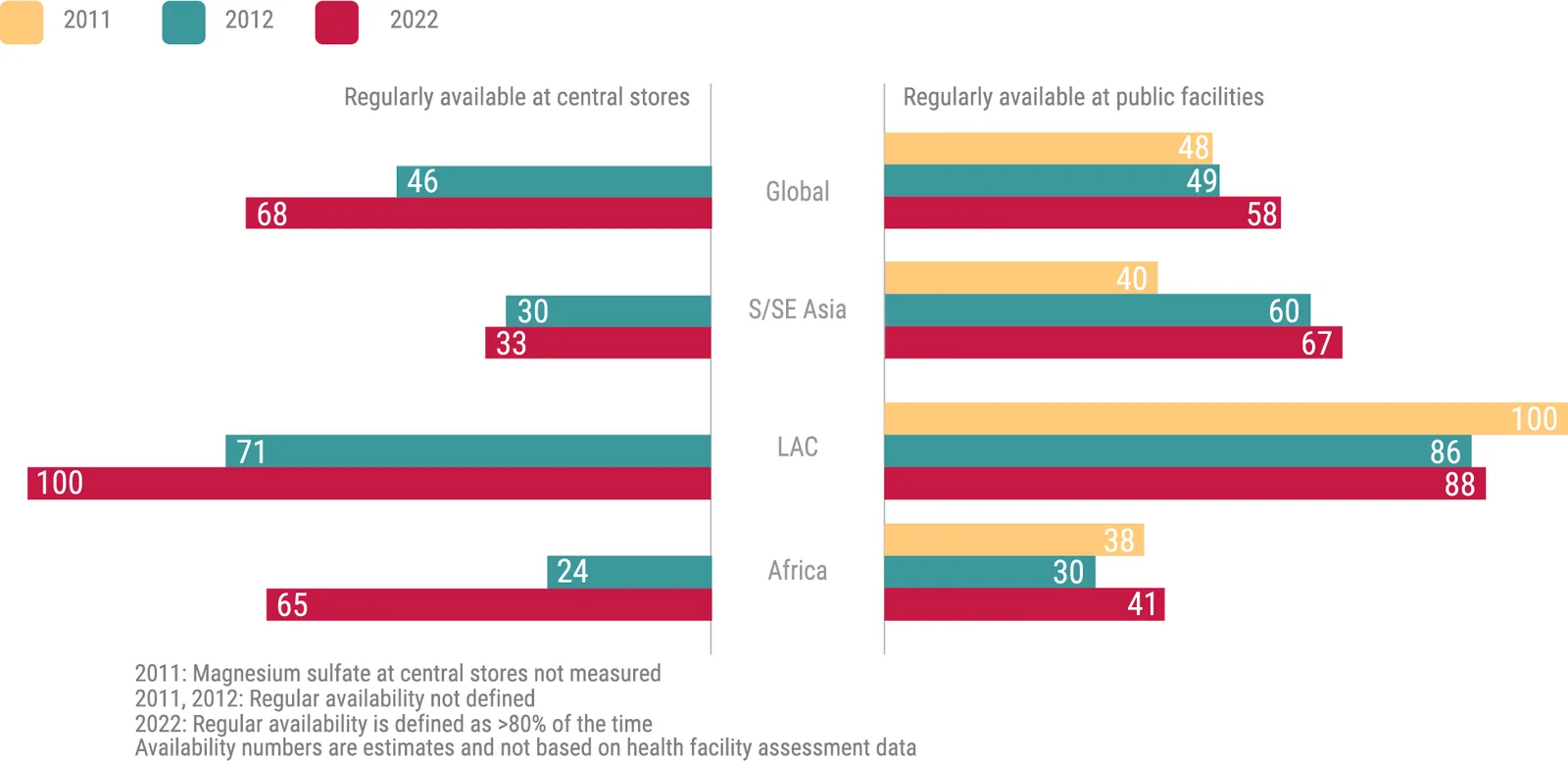
Percentage of countries estimating magnesium sulfate availability at central stores and public facilities, 2011-2022

For essential HDP drugs, availability has improved modestly since 2011. In 2022, 68% of countries reported that MgSO4 stockouts at central stores occurred rarely (i.e., once per year or less often), compared to 46% of countries in 2012. Regular availability of MgSO4 at public facilities rose slightly, reported by 49% of countries in 2012 and by 58% of countries in 2022 (See Fig. 5). Anti-hypertensive drug availability was not included in the 2011 or 2012 surveys.

**Fig. 6:**
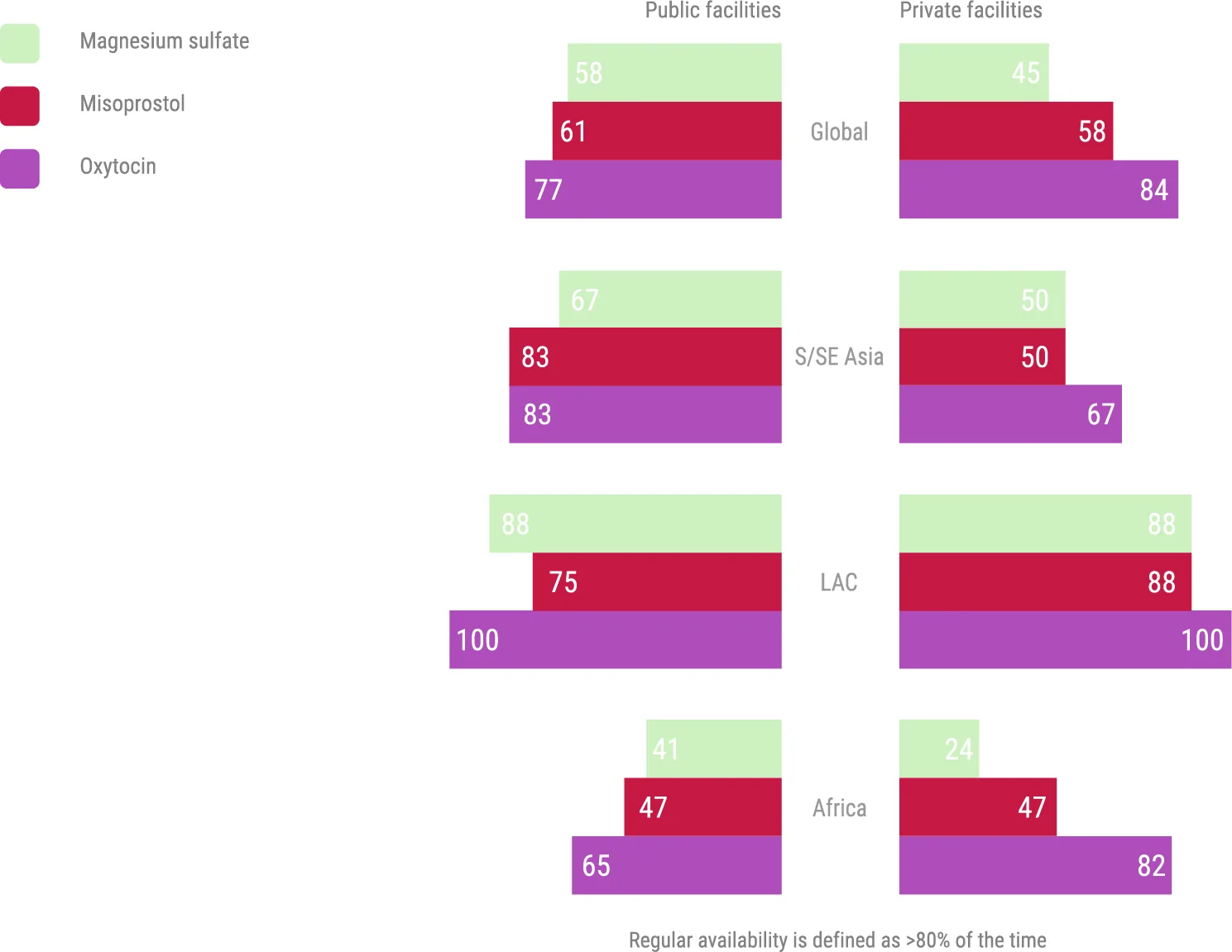
Percentage of countries estimating regular availability of essential PPH and HDP drugs at public and private facilities, 2022

In 2022, countries reported the availability of essential PPH and HDP drugs at the facility level as relatively similar between public and private facilities (See Fig. 6). They identified nifedipine as the most regularly available drug, with 65% of countries reporting it as available in public facilities and 52% of countries reporting it as available in private facilities. Hydralazine and labetalol were reported to be least regularly available, with 32% and 35% of countries estimating both hydralazine and labetalol to be regularly available at public and private facilities, respectively.

### National guidelines updated to incorporate the latest global standards

In assessing the reported inclusion of 23 current WHO guidelines for PPH and HDP drugs and practice, 61% of countries had a composite score of 80% or higher for PPH, and 39% for HDP.

All countries reported integration of three of the seven WHO recommendations for PPH into their national guidelines: (1) oxytocin as the preferred uterotonic; (2) a policy for safe blood transfusion; and (3) Active Management of the Third Stage of Labor (AMTSL) policy updated to include immediate use of an uterotonic. Inclusion of more recent WHO recommendations for PPH treatments—uterine balloon tamponade (UBT) and tranexamic acid—were reported by 90% and 77% of countries, respectively. Qualitative results indicated interest in scaling up UBT as a PPH treatment strategy, as many countries have some experience in using UBT. One country reported that:

*“Scale up of UBT [is] cost-effective*,* easy to use and assemble*,* and we [should] have available the balloon tamponade [for] all”- India national experts*

Recommended PPH interventions that were less frequently reported as included in national guidelines included heat-stable carbetocin for PPH prevention (reported by 45% of countries) and oxytocin injection in the umbilical vein for retained placenta (reported by 13% of countries).

Almost all countries (97%) reported having updated their national guidelines to include criteria for induction of labor before term in women with severe pre-eclampsia/eclampsia (PE/E). Many countries (84%) reported that national guidelines were updated to include the current WHO recommendations for short- and long-term management of women with HDP, preventative measures of giving low-dose aspirin during pregnancy for women with HDP risk factors, and calcium supplementation during pregnancy for women with low dietary calcium. All countries reported that at least one of the four WHO recommended antihypertensives to treat severe PE/E (hydralazine, labetalol, methyldopa, and nifedipine) was included in their national guidelines in 2022.

**Fig. 7:**
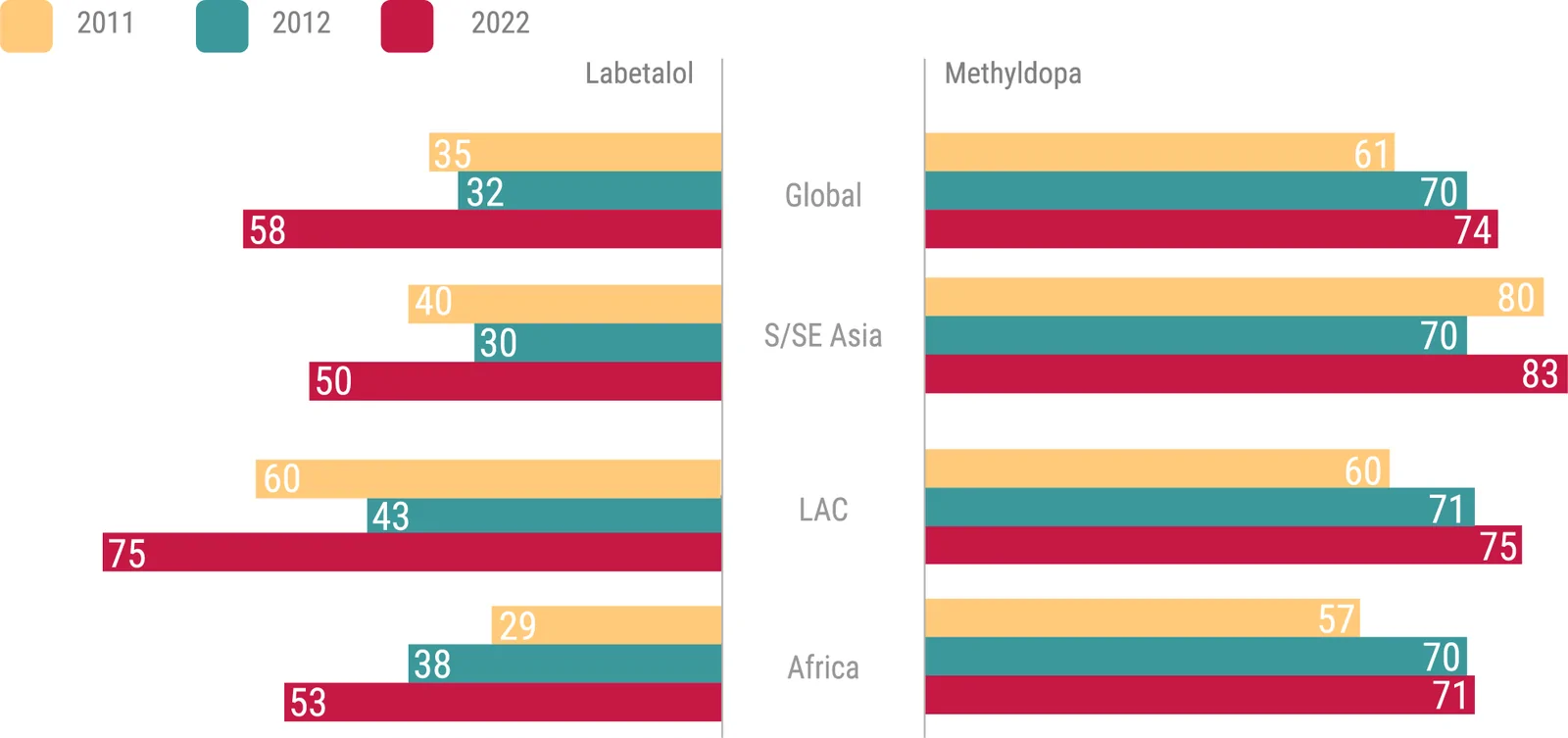
Percentage of countries estimating inclusion of WHO recommended anti-hypertensive drugs in the national guidelines, 2011-2022

Temporal comparative data for this theme were limited to inclusion of antihypertensive drugs to treat severe PE/E. More countries reported having included labetalol and methyldopa in their national guidelines since 2011 than nifedipine and hydralazine, the inclusion of which remained relatively constant over time. In 2011, 35% of countries reported including labetalol in their national guidelines, compared to 32% in 2012 and 58% in 2022. In 2011, 61% of countries reported including methyldopa in their national guidelines, rising to 70% in 2012 and 74% in 2022 (See Fig. 7).

Most countries reported that the private sector follows national guidelines with 90% of countries adhering to PPH guidelines and 77% of countries adhering to national guidelines for HDP, respectively. In the qualitative findings from the most recent survey, many countries identified improving use of national guidelines in the private and public sector as an opportunity for public-private collaboration. One country described the need for improved adherence to guidelines:

*“The majority of the private sector are not aware of the [national] guidelines*,* [there is] ignorance of their effectiveness*,* although some few try to use them*,* they are not consistent.”- Nigeria national experts*

### Medication quality and procurement policies at the national level

65% of countries had a composite score of 80% on this theme, a new addition to the 2022 survey. Almost all countries (94%) reported having policies in place for national procurement and distribution, storage, and transportation to ensure quality products according to WHO qualifications. Only 55% of countries reported that public facilities and 61% reported that private facilities maintain a controlled cold chain for oxytocin. For HDP, 71% of countries estimated that public facilities, and 58% estimated that private facilities, have a system in place to ensure availability of a correct 50% solution of MgSO4.

### Midwifery scope of practice

**Fig. 8:**
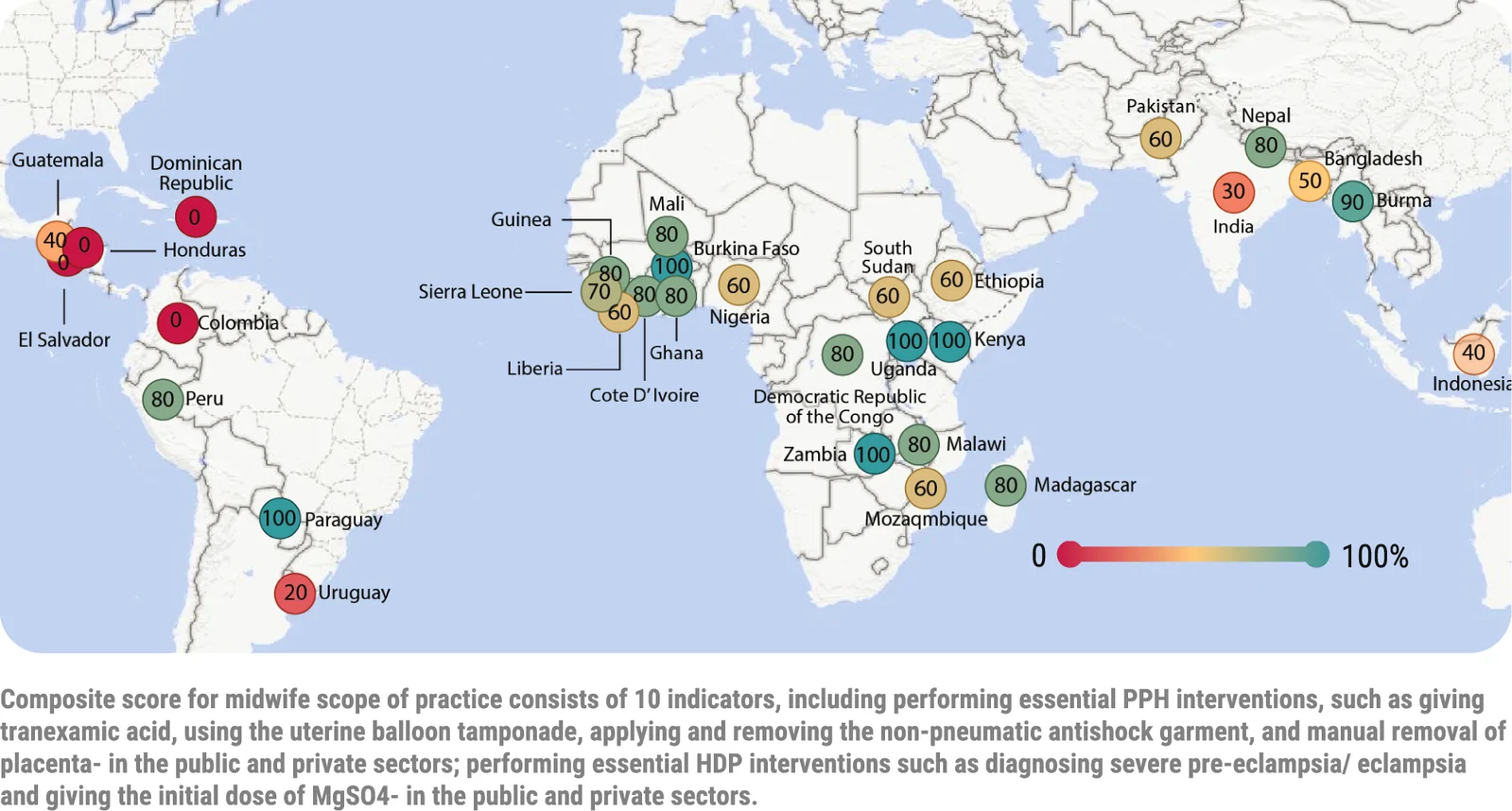
Overview of midwife scope of practice for PPH and HDP in 31 countries, 2022

This theme examined the scope of practice for formally trained midwives. Approximately half of surveyed countries (48%) scored 80% or higher on their composite score. Meaningful regional differences marked this theme, as some LAC countries reported not having a formally trained midwife cadre—though both Guatemala and the Dominican Republic report a midwife cadre is under development (Fig. 8). From 2011 to 2022, inclusion of two essential lifesaving skills in the scope of practice for formally trained midwives—manual removal of placenta and diagnosing severe PE/E and giving the first loading dose of MgSO4—did not improve. In 2011, 76% of countries reported that the midwifery scope of practice included manual removal of the placenta; and in 2022, this proportion was 77%. Diagnosing severe pre-eclampsia and giving the first loading dose of MgSO4 was reported as part of the midwifery scope by 86% of countries in 2011 and 81% in 2022.

In general, countries reported that the private sector had included fewer PPH interventions in the midwifery scope of practice than the public sector. This was particularly true for more recent global guidelines for PPH treatments, such as the use of tranexamic acid, UBT, and the non-pneumatic antishock garment. Across surveyed countries, 65% of countries reported that the public sector midwifery scope of practice included tranexamic acid and UBT, and 45% reported that it included the non-pneumatic antishock garment. In contrast, 61% of countries reported that the private sector midwifery scope included tranexamic acid, 52% included UBT, and 29% included the non-pneumatic antishock garment. Reasons for the differences in midwifery scope between the public and private sectors remain unclear, though qualitative findings suggest a possible gap in training:

*“Uterine balloon tamponade and applying the anti-shock garment are part of competencies of midwives but are not routinely practiced in the private sector facilities because of lack of training.”- Malawi national experts*

### Capacity building and training in global best practices

This theme looked at whether pre- and in-service curricula had been updated to current WHO recommendations on PPH and HDP in both public and private training institutions.

There were similar patterns in the data for both pre- and in-service curricula, with more countries reporting curricula updates for public training institutions than private institutions. All countries reported that public institutions were using updated global guidelines for quality-assured oxytocin as first-line uterotonic, AMTSL, and MgSO4 as first-line anticonvulsant for PE/E and management of severe hypertension in pregnancy. Countries least commonly reported that umbilical vein injection of oxytocin for the treatment of retained placenta had been integrated into pre- and in-service curricula for public and private institutions.

### National reporting on selected maternal and newborn health indicators

Since 2012, the proportion of countries reporting inclusion of a PPH and HDP indicator in the HMIS has increased. In 2022, 74% of countries reported including an indicator for PPH (use of a uterotonic immediately after birth for PPH prevention), compared to 43% in 2012. Similarly, 87% of countries reported including an indicator for HDP (number of women with severe PE/E) in 2022, compared to 51% in 2012.

Private sector figures were lower, with 45% of countries estimating that the private sector reports to HMIS on the PPH indicator (compared to 74% of countries for the public sector) and 52% of countries estimating the private sector reports on the HDP indicator (compared to 87% of countries for the public sector).

### Bottleneck and scale-up opportunities

Responses to qualitative questions on challenges and opportunities for scale-up of PPH and HDP national programs highlighted recurrent bottlenecks, including challenges related to drug procurement and distribution, poor quality of drugs at point of delivery, inadequate financing of the health system, insufficient human resources, inadequate capacity of MNH skilled workers, inadequate commodities, lack of public-private partnerships, need for improved data collection and data review systems, geographical challenges, and need for improved referral systems. The three recurring areas of focus that emerged from this analysis were public and private sector collaboration, quality assurance of medicines and commodities, and quality improvement approaches for management of these complications.

For public and private sector collaboration, national experts noted the need to improve systems to improve adherence to national guidelines and to strengthen capacity building and training on the PPH and HDP updated global guidelines:

*“The Ministry of Health and Social Welfare provides training in national guidelines and clinical updates to the private sector. Adherence to these national guidelines issued by the MOH is mandatory.”* – *Paraguay national experts*

*“[An opportunity for scale-up is the] introduction of evidence-based practices in private medical colleges*,* which would enhance quality of services of graduates*,* more skills-based management*,* mentoring and coaching and [introducing] innovations and expansion of innovations (NASG*,* condom tamponade)*,* and manufacturing through the private sector.”* - *Nepal national experts*

National experts also suggested the need to improve the referral system within and between public and private sectors, as well as monitoring and evaluation efforts across sectors:

*“[There is] a weak referral system. There needs to be an affordable and sustainable referral system.”* – *Sierra Leone national experts*

*“[We need] accountability*,* monitoring*,* evaluation and supervision by the MOH to providers of maternal and neonatal services [in public and private sectors].”* – *Honduras national experts*

National experts also observed several bottlenecks impeding quality assurance and improvement in commodities and practices, including improving the quality of care and quality assurance for medications. Suggestions for opportunities included:

*“Standardization [of education programs] with on-site simulation*,* ongoing supervision and creating of tele-assistance networks between hospitals.”* – *Colombia national experts*

*“Increase[ing] the budget allocation for supplies for maternal and newborn health and make[ing] MgSO4 freely available at all facilities.”* - *Madagascar national experts*

Experts emphasized the importance of a policy environment that supports quality assurance of medicines and commodities, as well as processes aimed at improving the management of complications at hospitals.

*“[Provide] clarity on management protocols irrespective of existing service delivery guidelines.”* - *India national experts*

## Discussion

These assessment findings come at a pivotal time, aligning with recent strategic planning frameworks developed by the Every Newborn Action Plan/Ending Preventable Maternal Mortality initiative and the global Roadmap to Combat Postpartum Hemorrhage. Additionally, the groundbreaking results from the recent E-MOTIVE trial hold direct implications for maternal health programs [10]. E-MOTIVE demonstrated the efficacy of early PPH detection through a bundled treatment approach including uterine massage, oxytocic drugs, tranexamic acid, intravenous fluids, thorough examination, and escalation if necessary. Implementation of this bundle resulted in reductions of up to 60% in maternal mortality, blood loss, and the need for laparotomy (surgery for bleeding) [10].

Our study uncovered several compelling trends in scaling up PPH and HDP programs across the 31 surveyed countries. Over the last decade, most countries have aligned their policy documents with current global standards, encompassing EMLs, national clinical guidelines, and pre- and in-service curricula for providers of obstetric complications. Since 2011, both oxytocin and MgSO4—essential drugs for PPH and HDP—have consistently been included in all countries’ EMLs and national guidelines. Uptake of other interventions has been uneven across policy, national guidelines, and curricula. For instance, misoprostol adoption for PPH treatment and prevention appears to have increased significantly. Misoprostol is reported in nearly all countries’ national policies and since 2012, increased from 12% to 60% for countries reporting it is regularly available at the facility level. However, uptake of tranexamic acid and the non-pneumatic antishock garment have been limited. Similarly, implementation of low-dose aspirin for high-risk women and calcium supplementation for those in areas with low dietary calcium intake to prevent HDP has also been slow. Uptake of newer interventions like UBT and umbilical vein injection is limited, most likely because these interventions are only recommended in certain contexts that meet several conditions [11].

Hypertensive disorders of pregnancy remain a leading cause of maternal death with long-term cardiovascular risks for the mother [4, 19]. Additionally, HDP can also cause higher perinatal mortality when babies are born too early and require special care, as many LMICs have limited access to the recommended neonatal intensive care interventions needed to save these lives [5, 19]. The policy environment reflects global evidence for HDP; the majority of countries (87%) have incorporated short- and long-term management of women with HDP into their national guidelines. However, limited progress has been made over the last decade to improve availability of first-line anticonvulsant medication at the facility level, with only 58% and 45% of countries reporting that MgSO4 is regularly available in public facilities and private facilities, respectively. Qualitative responses about bottlenecks highlighted the importance of addressing adequate drug availability, competence of MNH skilled personnel, and data collection and management for HDP through the HMIS in both public and private sectors. Coordinated efforts are required to improve the management of hypertensive disorders of pregnancy and mitigate long-term risks [4, 19].

The private sector plays a significant role in providing maternity care, with a recent review of low- and middle-income countries revealing that the private sector provides more than one-third of maternal health services, accounting for a 44% mean market share of antenatal care clients, and a 40% mean market share for delivery care [17, 18]. While historical data from the private sector is limited, countries reported greater variation in clinical practices in the private sector compared to the public sector. Several national experts in the 2022 survey identified building and strengthening public-private partnerships as a priority, noting that opportunities included collaborating on maternal perinatal death surveillance and response, involvement in regular data review and work planning processes, and greater private sector representation on decision-making committees. Optimizing engagement with the private sector, alongside improved governance and regulation, will be critical in national and global efforts to end preventable maternal deaths [18, 20]. Furthermore, the relatively low proportions of countries reporting functional cold chains for oxytocin in public and private facilities, or systems ensuring the procurement of correctly concentrated MgSO4 solutions, highlights the urgent need to enhance quality assurance across the entire procurement, supply, and distribution pathway up to the point of delivery.

Problematically, there have been regulatory and practice impediments that limit midwives’ ability to practice the full scope of their education. An example is reflected in the limited change in the inclusion of two essential lifesaving skills—manual removal of placenta and giving the loading dose of MgSO4—between the 2011 and 2022 surveys. Although the International Confederation of Midwives has articulated well-defined competencies and midwifery is considered an essential cadre by the WHO, our data suggests that midwifery practice often falls short of these competency frameworks [21, 22]. When midwives are trained to practice to their full scope, they can provide better quality and more comprehensive services. Additionally, midwives’ ability to provide high-quality care is contingent upon an enabling work environment and a functional health system with adequate commodities and supplies and functional referral systems [23].

**Fig. 9:**
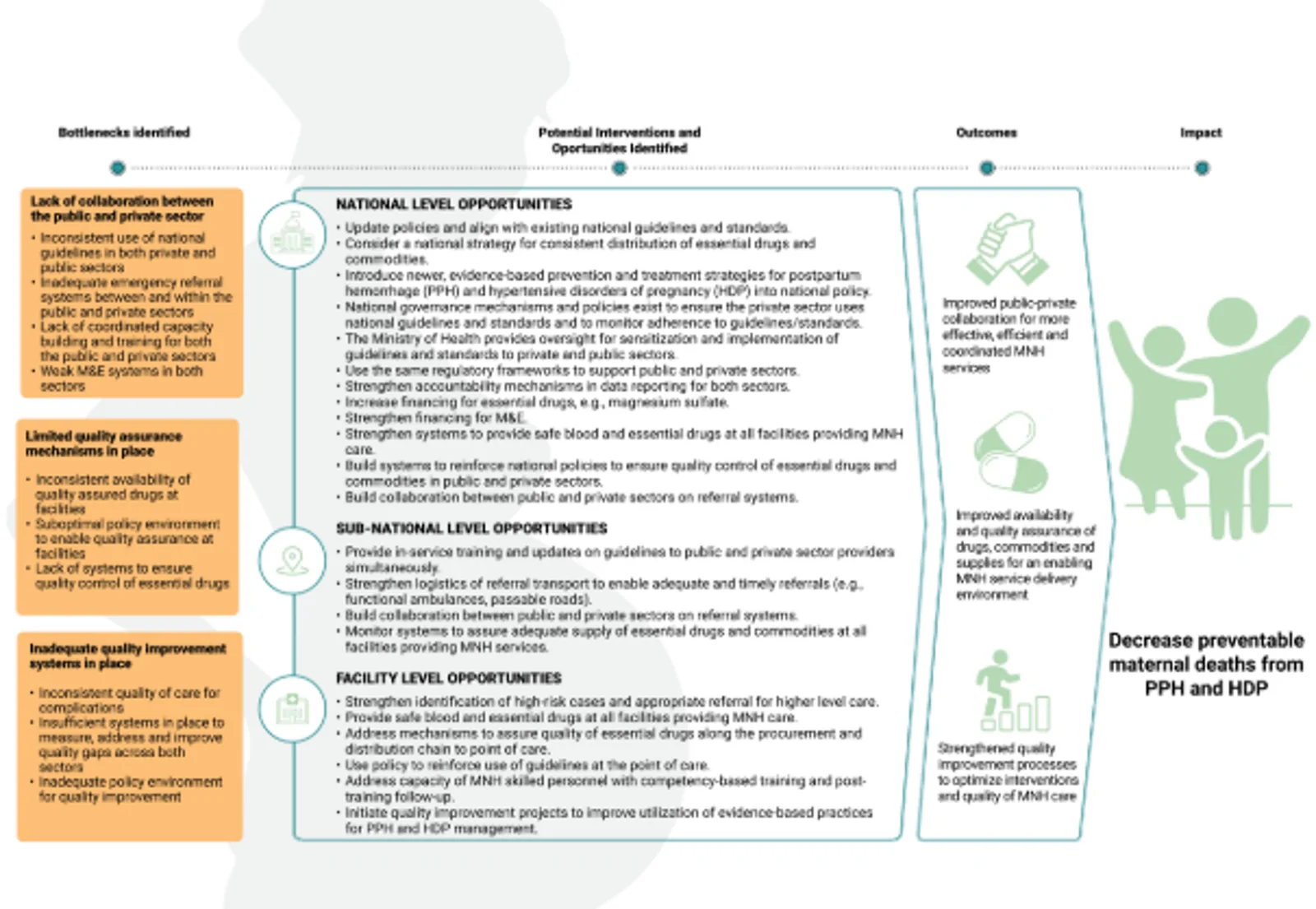
Bottlenecks and opportunities for scaling up PPH and HDP programs

Based on the findings presented in this manuscript, recommendations are warranted in several key areas: improved public-private collaboration, assurance of an enabling clinical environment, availability of quality-assured life-saving medicines where they are needed, and system-wide quality improvement processes that are implemented at national scale. Opportunities to improve quality of care for PPH and HDP exist at all levels of the health system across both public and private sectors (Fig. 9).

Nationally, these opportunities include prioritizing integration of recent global updates for PPH and HDP into national policies, guidelines, and pre- and in-service training curricula. Engaging the private sector and professional associations in the revision and updating of policy documents, national guidelines, and curricula may be helpful to promote collaboration across sectors, leading to improved quality and better coordination of care. Improving the availability and quality of essential medicines and commodities will demand strengthened policies and focused action to increase availability at lower levels, prioritize WHO pre-qualified manufacturers, and improve logistics distribution systems to facilities. This may lead to consistent availability and timely utilization of lifesaving medications at the point of care, in line with recommendations outlined in the UN Commission on Life-Saving Commodities for Women and Children [24].

Collaboration with the private sector is also crucial to ongoing efforts to prevent maternal and newborn deaths, particularly in areas such as skilled workforce training, logistics and commodity management, monitoring, learning and evaluation, strategic planning, and standardization of clinical practice guidelines. Recent studies have also emphasized that successfully engaging the private sector to improve maternal and newborn outcomes will require overall health systems strengthening approaches, including financing, regulation, and capacity building of governments to steward mixed health systems [20].

Furthermore, in facilities where midwives practice, it is crucial that their scope of practice includes management of basic obstetric emergencies so that they can deliver lifesaving services. Attention to expanding the midwifery scope of practice and focusing on competency-based skills training in accordance with the skills and core competencies set forth by the International Confederation of Midwives would optimize their potential to save lives [21, 22].

Last, sustained efforts are needed to improve existing indicators; streamline data collection methods; improve measurement systems and data quality for adaptive management; and strengthen regulatory and accountability mechanisms. Improving all sector reporting in the HMIS, especially from the private sector, and improving accountability mechanisms for HMIS reporting will enhance the quality and accuracy of data captured at the facility level. In addition, using an indicator that reflects treatment and management for PE/E will be more informative than an indicator reflecting diagnosis alone. All of these are important measurement priorities for the Ending Preventable Maternal Mortality initiative and the Every Newborn Action Plan [25, 26].

Our study has several strengths and limitations. This assessment was conducted in multiple countries using a standardized methodology that was refined from our experiences with the previous rounds of the survey [6]. We used an assessment tool developed through an international collaborative process to assess these maternal health complications comprehensively. The qualitative data enriched the quantitative findings and provided nuanced insights and helped triangulate study findings. However, despite our efforts to minimize bias, this survey also had several limitations. First, there may have been other context-specific differences between public and private sectors: for example, we asked questions on national guidelines, but this may not accurately reflect routine clinical practice. More broadly, the findings reflect expert consensus and self-reported national data rather than direct assessment of policy implementation or facility practice and should therefore be interpreted as indicative of the national policy and programme environment and confirmed through facility-based assessment before conclusions are drawn about actual service delivery. Second, the survey was implemented during the COVID-19 pandemic, which required flexibility in data collection to accommodate local protocols for mitigation of COVID-19. Third, local variation in access to information such as records on commodity stocks during the consultative meeting may have caused some countries to respond differently and ultimately introduce subjectivity to estimation instead of verifying responses with national data or facility level assessments. Fourth, social desirability bias could have positively influenced the findings, as the national focal person who convened the expert groups was associated with either the MOMENTUM project or UNFPA. Discrepant responses among informants were discussed in consultative meetings and reconciled against nationally relevant documents before a single consensus response was submitted, though the degree of documentary verification and the disciplinary mix of informants varied across countries. Qualitative coding was undertaken by a single researcher with study-team review, which may introduce interpretive bias.

Last, we did not assess the validity of the different composite scores that were used to analyze the data because this was beyond the scope of the current study. The scores also gave equal weight to all indicators and should therefore be interpreted as broad, comparative indicators rather than precise measures of programme performance. However, despite these study limitations, we believe that this study is important to guide advocacy and drive planning and action in participating countries.

## Conclusions

This multi-country assessment spanning 31 countries across sub-Saharan Africa, South and Southeast Asia, and Latin America and the Caribbean, and examining both public and private sectors; provides one of the few comparative views of how national programs for PPH and HDP have evolved over a decade. The findings show clear progress in aligning policies, EMLs, and guidelines with global standards, alongside persistent gaps that converge on a few priorities: inconsistent facility-level availability of essential commodities, most notably MgSO4; slow uptake of newer interventions such as tranexamic acid; a midwifery scope of practice that still excludes key lifesaving skills; and weak quality assurance and data reporting, particularly in the private sector. Addressing these gaps will require coordinated action to integrate updated guidelines, strengthen procurement and supply chains, expand competency-based midwifery practice, and improve private-sector governance and HMIS reporting. By identifying where implementation lags policy across diverse health systems, this assessment offers a practical evidence base to guide advocacy, planning, and accountability under the Ending Preventable Maternal Mortality initiative and the Every Newborn Action Plan, and to inform future efforts to reduce maternal mortality from PPH and HDP.

## Supplementary Information

Supplementary Material 1.

Supplementary Material 2.

## Data Availability

All data generated or analyzed during this survey are included in this published article and its supplementary information files. The detailed presentation on which the manuscript is based is available in the public domain. https://decfinder.devme.ai/usaid-momentum/momentum-pph-and-hdp-survey-preliminary-findings-2022/.

## Abbreviations

AMTSL: Active Management of the Third Stage of Labor
EML: Essential Medicines List
HDP: Hypertensive Disorders of Pregnancy
HMIS: Health Management Information System
LAC: Latin America and the Caribbean
MgSO4: Magnesium Sulfate
MMR: Maternal Mortality Ratio
MNH: Maternal and Newborn Health
MOH: Ministry of Health
NASG: Non-Pneumatic Anti-Shock Garment
PE/E: Pre-Eclampsia/Eclampsia
PPH: Postpartum Hemorrhage
SDG: Sustainable Development Goal
TXA: Tranexamic Acid
UBT: Uterine Balloon Tamponade
UNFPA: United Nations Population Fund
USAID: United States Agency for International Development
WHO: World Health Organization
