# Supplementary material for: Prevention and management of postpartum hemorrhage and hypertensive disorders of pregnancy: results from a 31-country rapid assessment
Source: BMC Pregnancy Childbirth. 2026 Jul 20;26:1191. doi: 10.1186/s12884-026-09679-w (PMC13681778; doi:10.1186/s12884-026-09679-w)
Supplement: Supplementary file 1 [file 12884_2026_9679_MOESM1_ESM.docx]

# Additional File 1 – Multi-country analysis Survey Tool

Thank you for participating in the multi-country analysis on postpartum hemorrhage and hypertensive disorders of pregnancy policies, practices and commodities in the public and private sectors.

- This survey reflects the most current updates and guidelines on [PPH](https://www.jhpiego.org/wp-content/uploads/2020/07/Updated-WHO-PPH-recommendations_Widmer_FINAL.pdf) and [HDP](https://www.who.int/reproductivehealth/publications/maternal_perinatal_health/9789241548335/en/) of the World Health Organization;
- In this survey "private sector" refers to all facilities or institutions that are beyond the purview of national governments that includes for-profit, not-for-profit, faith-based and other charitable institutions;
- Each key informant will need to read and sign the [informed consent](https://www.surveymonkey.com/r/LMJS5RY);
- It is possible to change responses until the survey deadline of 31 March 2022;
- Please refer to national policies, service delivery guidelines, and other national documents to verify responses;
- If you have any technical difficulties, please contact Mathea Pielemeier via email at [Mathea.Pielemeier@jhpiego.org.](mailto:Mathea.Pielemeier@jhpiego.org)

#### Thank you for your important contributions to better understand current progress in postpartum hemorrhage and hypertensive disorders of pregnancy.

### Section I: Postpartum Hemorrhage (PPH)

### Policy

1. Are either of the following medications on the National Essential Medicine List (EML) for **prevention and treatment** of PPH?

|  | Yes | No |
| --- | --- | --- |
| Oxytocin | 🞏 | 🞏 |
| Misoprostol | 🞏 | 🞏 |

2. Is tranexamic acid on the EML for **treatment** of PPH?

🞏 Yes	🞏 No

3. Is heat-stable carbetocin on the EML for the **prevention** of PPH?

🞏 Yes	🞏 No

4. Are the PPH service delivery guidelines used in the private sector consistent with national service delivery guidelines?

🞏 Yes	🞏 No

Please list any additional comments here:

5. Do the national service delivery guidelines include **oxytocin** as preferred uterotonic?

🞏 Yes	🞏 No

6. Are national service delivery guidelines updated to include the following PPH treatments?
   Select all that apply:

|  | Yes | No |
| --- | --- | --- |
| Tranexamic acid | 🞏 | 🞏 |
| Uterine balloon tamponade | 🞏 | 🞏 |
| Non-pneumatic anti-shock garment | 🞏 | 🞏 |
| Umbilical vein injection of oxytocin retained placenta | 🞏 | 🞏 |

7. Is it national policy to offer women delivering at home or without a skilled birth attendant, **misoprostol** to be taken after birth for PPH prevention?

🞏 Yes	🞏 No

8. Are there relevant national procurement policies for the medications listed below that assure quality products (WHO qualified or by relevant national authority) as they are procured? Select all that apply:

|  | Yes | No |
| --- | --- | --- |
| Oxytocin | 🞏 | 🞏 |
| Misoprostol | 🞏 | 🞏 |
| Ergometrine | 🞏 | 🞏 |

Please list any additional comments here:

9. Are there policies in place for the below medications' distribution, storage, and transport (from port to facility) that meet international norms? Select all that apply

|  | Yes | No |
| --- | --- | --- |
| Oxytocin | 🞏 | 🞏 |
| Misoprostol | 🞏 | 🞏 |
| Ergometrine | 🞏 | 🞏 |

Please list any additional comments here:

10. What medications are approved through national policy and service delivery guidelines for PPH prevention if quality of oxytocin cannot be guaranteed? Select all that apply

🞏 Heat-stable carbetocin

🞏 Ergometrine/methylergometrine

🞏 Oxytocin-ergometrine

🞏 Misoprostol

11. Is there a national policy for safe blood transfusion services?

🞏 Yes	🞏 No

12. Is national policy for Active Management of the Third Stage of Labor updated to include immediate use of a uterotonic?

🞏 Yes	🞏 No

13. Do midwives' scope of practice include (select all that apply):

|  | Public sector  midwives | Private sector  midwives |
| --- | --- | --- |
| Giving tranexamic acid | 🞏 | 🞏 |
| Management of uterine balloon tamponade | 🞏 | 🞏 |
| Performing umbilical vein injection | 🞏 | 🞏 |
| Applying/removing non- pneumatic anti-shock garment | 🞏 | 🞏 |
| Performing manual removal of placenta | 🞏 | 🞏 |

Please list any additional comments here:

14. Are any of the professional societies listed below involved in development and updating of national level PPH documents (e.g., national policies, service delivery guidelines, pre-service and in-service curricula, and/or quality assurance and quality improvement)?

|  | Yes | No |
| --- | --- | --- |
| Midwifery | 🞏 | 🞏 |
| Nursing | 🞏 | 🞏 |
| Obstetrics/gynaecology | 🞏 | 🞏 |

Other (please specify) :

### CAPACITY BUILDING

15. Are **pre-service** curricula for training institutions updated for all skilled maternal newborn health (MNH) personnel to include (select all that apply)

|  | Public training institutions | Private training institutions |
| --- | --- | --- |
| Oxytocin preferred uterotonic if quality can be assured, otherwise other 1st line uterotonic recommended | 🞏 | 🞏 |
| Tranexamic acid included as treatment for PPH | 🞏 | 🞏 |
| Uterine balloon tamponade as 2nd line treatment of PPH due to atony in settings with appropriate context | 🞏 | 🞏 |
| Non-pneumatic anti-shock garment for 2nd line  treatment of PPH | 🞏 | 🞏 |
| Umbilical vein injection of oxytocin for treatment of retained placenta | 🞏 | 🞏 |
| Updated Active Management of the Third Stage of Labor (AMTSL). | 🞏 | 🞏 |

Please list any additional comments here:

16. Are **in-service** curricula for training institutions updated for all skilled maternal newborn health (MNH) personnel to include (select all that apply):

|  | Public training institutions | Private training institutions |
| --- | --- | --- |
| Oxytocin preferred uterotonic if quality can be assured, otherwise other 1st line uterotonic recommended | 🞏 | 🞏 |
| Tranexamic acid included as treatment for PPH | 🞏 | 🞏 |
| Uterine balloon tamponade as 2nd line treatment of PPH due to atony in settings with appropriate context | 🞏 | 🞏 |
| Non-pneumatic anti-shock garment for 2nd line  treatment of PPH | 🞏 | 🞏 |
| Umbilical vein injection of oxytocin for treatment of retained placenta | 🞏 | 🞏 |
| Updated Active Management of the Third Stage of Labor (AMTSL). | 🞏 | 🞏 |

Please list any additional comments here:

17. Which private sector providers are included in public sector in-service training on PPH?
    Select all that apply:

🞏 From non-governmental organizations From faith-based organizations

🞏 From for-profit organizations

🞏 Don’t know

🞏 Other (please specify):

### LOGISTICS

18. Is **oxytocin** regularly available at facilities that offer maternity services?

|  | Regularly (>80% of time) | More than half  the time  (50-80% of time) | Less than half  the time  (<50% of time) | Procured externally by patient/family | Never |
| --- | --- | --- | --- | --- | --- |
| Public facilities | 🞏 | 🞏 | 🞏 | 🞏 | 🞏 |
| Private facilities | 🞏 | 🞏 | 🞏 | 🞏 | 🞏 |

Please list any additional comments here:

19. Is **tranexamic acid** regularly available at facilities that offer maternity services?

|  | Regularly (>80% of time) | More than half  the time  (50-80% of time) | Less than half  the time  (<50% of time) | Procured externally by patient/family | Never |
| --- | --- | --- | --- | --- | --- |
| Public facilities | 🞏 | 🞏 | 🞏 | 🞏 | 🞏 |
| Private facilities | 🞏 | 🞏 | 🞏 | 🞏 | 🞏 |

Please list any additional comments here:

20. Is **misoprostol** regularly available at facilities that offer maternity services?

|  | Regularly (>80% of time) | More than half  the time  (50-80% of time) | Less than half  the time  (<50% of time) | Procured externally by patient/family | Never |
| --- | --- | --- | --- | --- | --- |
| Public facilities | 🞏 | 🞏 | 🞏 | 🞏 | 🞏 |
| Private facilities | 🞏 | 🞏 | 🞏 | 🞏 | 🞏 |

Please list any additional comments here:

21. Do **health** facilities that offer maternity services have a controlled cold-chain (refrigeration at the correct temperature) for oxytocin prior to use?

|  | Yes | Sometimes | No |
| --- | --- | --- | --- |
| Public facilities | 🞏 | 🞏 | 🞏 |
| Private facilities | 🞏 | 🞏 | 🞏 |

22. Is **oxytocin** free of charge to patients at facilities that offer maternity services?

|  | Yes | Sometimes | No |
| --- | --- | --- | --- |
| Public facilities | 🞏 | 🞏 | 🞏 |
| Private facilities | 🞏 | 🞏 | 🞏 |

23. How frequently do stock-outs of **oxytocin** occur?

|  | Frequently (once in every  2 months) | Sometimes (every 3-6 months) | Rarely  (once a year) |
| --- | --- | --- | --- |
| At the central level | 🞏 | 🞏 | 🞏 |
| At the district/regional level | 🞏 | 🞏 | 🞏 |

Please list any additional comments here:

24. Is there a logistics management system independent of the national procurement system that private sector facilities use to procure essential medications?

🞏 Yes	🞏 No

25. From where does the private sector source PPH commodities? Select all that apply:

🞏 Received free from the government central medical store

🞏 Received free from donors

🞏 Procured from local wholesaler/retailers

🞏 Other (please specify):

26. How does the private sector receive and distribute PPH commodities? Select all that apply:

🞏 Government deliveries

🞏 Private sector goes to source to collect

🞏 Private sector has wholesaler/ other distributor delivery

🞏 Other (please specify):

### NATIONAL REPORTING OF MH INDICATOR

27. Is an indicator of giving a uterotonic immediately after delivery included in the national Health Management Information System (HMIS)?

🞏 Yes	🞏 No

28. Does the private sector report on uterotonic use immediately after delivery on the national HMIS?

🞏 Yes	🞏 No

If no, why not?:

29. Are there national level accountability mechanisms that link reporting of the national HMIS indicator to implementation of the policy and guidelines for PPH prevention and treatment at the facility level?

🞏 Yes	🞏 No

If yes, please describe:

### PROGRAMMING

30. Which activities in PPH prevention and management are being supported by the Ministry of Health (MOH), World Health Organization (WHO), UNICEF, UNFPA, and/or other multilateral implementing partners?
    Select all that apply:

🞏 Health system governance (health service financing, eliminating policy barriers)

🞏 National policy (essential medicine list, national policies and service delivery guidelines consistent with current WHO standards)

🞏 Drugs and equipment (logistics, procurement, quality assurance)

🞏 Service delivery capacity at sites (reliable infrastructure, personnel and systems to deliver services)

🞏 Health worker pre-service and in-service training (competency based, curricula consistent with WHO)

🞏 Partnership development (private/public sector, professional associations, local government,
non-governmental organizations, MOH collaboration)

🞏 Community awareness of maternal newborn health issues (civil society, women’s group advocacy for early recognition of problems and referral)

🞏 Health technology and information systems (improved devices, procedures, systems of data management)

🞏 Other (please specify):

31. Which activities in PPH prevention and management are being undertaken by the private sector?
    Select all that apply:

🞏 Health system governance (health service financing, eliminating policy barriers)

🞏 National policy (essential medicine list, national policies and service delivery guidelines consistent with current WHO standards)

🞏 Drugs and equipment (logistics, procurement, quality assurance)

🞏 Service delivery capacity at sites (reliable infrastructure, personnel and systems to deliver services)

🞏 Health worker pre-service and in-service training (competency based, curricula consistent with WHO)

🞏 Partnership development (private/public sector, professional associations, local government, non-governmental organizations, MOH collaboration)

🞏 Community awareness of maternal newborn health issues (civil society, women’s group advocacy for early recognition of problems and referral)

🞏 Health technology and information systems (improved devices, procedures, systems of data management)

🞏 Other (please specify):

32. What percent of districts are covered by current national PPH programs? (Provide your best possible estimate)

🞏 90-100%

🞏 80-90%

🞏 70-80%

🞏 60-70%

🞏 50-60%

🞏 Less than 50%

### OPPORTUNITIES FOR EXPANSION AND SCALE-UP

33. Please describe any potential opportunities that you see for program introduction, expansion or scale-up in the private and public sectors.

34. What opportunities are there for the public and private sector to collaborate on PPH prevention and management? Select all that apply:

🞏 District work planning Regular data review

🞏 Maternal death audits

🞏 Civil society initiatives to reduce PPH

🞏 Other (please specify):

35. What are the three most significant bottlenecks to scaling up PPH reduction programs in your country? Briefly describe what is being done or could be done to address the bottlenecks.

1)

2)

3)

### Section II: Hypertensive disorders of pregnancy (HDP) POLICY

36. What drugs are approved through national policy/ service delivery guidelines for administration as
    first-line anti-hypertensives in **non-severe hypertension of pregnancy**? Select all that apply:

🞏 Beta blocker, e.g. labetolol

🞏 Hydralazine

🞏 Methyldopa

🞏 Nifedipine

🞏 Other (please specify):

37. What drugs are approved through national policy/ service delivery guidelines for administration as
    first-line anti-hypertensives in **severe pre-eclampsia or eclampsia**? Select all that apply:

🞏 Labetolol

🞏 Hydralazine

🞏 Methyldopa

🞏 Nifedipine

🞏 Other (please specify):

38. What drugs are listed on the National Essential Medicines List (EML), as anti- hypertensives in management of **severe pre-eclampsia or eclampsia**? Select all that apply:

🞏 Labetolol

🞏 Hydralazine

🞏 Methyldopa

🞏 Nifedipine

🞏 Other (please specify):

39. What drugs are approved through national policy/service delivery guidelines as first-line anticonvulsants for **severe pre-eclampsia or eclampsia**? Select all that apply:

🞏 Magnesium sulfate

🞏 Diazepam

🞏 Other (please specify):

40. Is magnesium sulfate on the National Essential Medicine List for **severe pre-eclampsia and eclampsia**?

🞏 Pre-eclampsia

🞏 Eclampsia

41. Do the national policy/service delivery guidelines include recommendations on induction of labor versus expectant management of women with **severe pre- eclampsia** before term?

🞏 Yes	🞏 No

42. Do the national policy/service delivery guidelines include recommendations on induction of labor versus expectant management of women with **mild pre- eclampsia** before term?

🞏 Yes	🞏 No

43. Do the national policy/service delivery guidelines include recommendations for short- and long-term management of women who have had HDP after giving birth?

🞏 Yes	🞏 No

44. Do the national policy/ service delivery guidelines include **low dose acetylsalicylic acid** for the prevention of pre-eclampsia in high-risk women?

🞏 Yes	🞏 No

45. Do the national policy/ service delivery guidelines include **calcium supplementation** in pregnancy for women living in areas of low dietary calcium?

🞏 Yes	🞏 No

46. Are the HDP service delivery guidelines used in the private sector consistent with national service delivery guidelines?

🞏 Yes	🞏 No

47. Are midwives authorized to diagnose severe pre-eclampsia/ eclampsia and administer initial (loading) dose of magnesium sulfate at the lowest level facility that they work at within the health system?

|  | Yes | No |
| --- | --- | --- |
| Midwives in the public sector | 🞏 | 🞏 |
| Midwives in the private sector | 🞏 | 🞏 |

48. Are any of the professional societies listed below involved in development and updating of national level HDP documents (e.g. national policies, service delivery guidelines, pre- service and in-service curricula, and/or quality assurance and quality improvement)?

|  | Yes | No |
| --- | --- | --- |
| Midwifery | 🞏 | 🞏 |
| Nursing | 🞏 | 🞏 |
| Obstetrics/gynaecology | 🞏 | 🞏 |

### CAPACITY BUILDING

49. Are **pre-service** curricula for training institutions updated for all skilled maternal newborn health (MNH) personnel to include (select all that apply):

|  | Public training institutions | Private training institutions |
| --- | --- | --- |
| Management of non-severe hypertension of pregnancy with methyldopa or beta blocker | 🞏 | 🞏 |
| Management of severe hypertension of pregnancy with labetalol, hydralazine, nifedipine, methyldopa | 🞏 | 🞏 |
| Anti-convulsant management of severe pre-eclampsia or eclampsia with MgSO4 as first line treatment | 🞏 | 🞏 |
| Use of daily low dose aspirin for the prevention of  pre-eclampsia in high-risk pregnant women | 🞏 | 🞏 |
| Calcium supplementation for pregnant women in areas of low dietary calcium for the prevention of pre-eclampsia | 🞏 | 🞏 |

Please list any additional comments here:

50. Are **in-service** curricula for training institutions updated for all skilled maternal newborn health (MNH) personnel to include (select all that apply):

|  | Public training institutions | Private training institutions |
| --- | --- | --- |
| Management of non-severe hypertension of pregnancy with methyldopa or beta blocker | 🞏 | 🞏 |
| Management of severe hypertension of pregnancy with labetalol, hydralazine, nifedipine, methyldopa | 🞏 | 🞏 |
| Anti-convulsant management of severe pre-eclampsia or eclampsia with MgSO4 as first line treatment | 🞏 | 🞏 |
| Use of daily low dose aspirin for the prevention of  pre-eclampsia in high-risk pregnant women | 🞏 | 🞏 |
| Calcium supplementation for pregnant women in areas of low dietary calcium for the prevention of pre-eclampsia | 🞏 | 🞏 |

Please list any additional comments here:

51. Which private sector providers are included in public sector in-service training on HDP? Select all that apply:

🞏 From non-governmental organizations From faith-based organizations

🞏 From for-profit organizations

🞏 Don’t know

🞏 Other (please specify):

### LOGISTICS

52. Is **magnesium sulfate** available at facilities that offer maternity services?

|  | Regularly (>80% of time) | More than half  the time  (50-80% of time) | Less than half  the time  (<50% of time) | Procured externally by patient/family | Never |
| --- | --- | --- | --- | --- | --- |
| Public facilities | 🞏 | 🞏 | 🞏 | 🞏 | 🞏 |
| Private facilities | 🞏 | 🞏 | 🞏 | 🞏 | 🞏 |

Please list any additional comments here:

53. Are the below medications available at public facilities that offer maternity services?

|  | Regularly (>80% of time) | More than half  the time  (50-80% of time) | Less than half  the time  (<50% of time) | Procured externally by patient/family | Never |
| --- | --- | --- | --- | --- | --- |
| Labetolol | 🞏 | 🞏 | 🞏 | 🞏 | 🞏 |
| Hydralazine | 🞏 | 🞏 | 🞏 | 🞏 | 🞏 |
| Nifedipine | 🞏 | 🞏 | 🞏 | 🞏 | 🞏 |
| Methyldopa | 🞏 | 🞏 | 🞏 | 🞏 | 🞏 |

Please list any additional comments here:

54. Are the below medications available at **private** facilities that offer maternity services?

|  | Regularly (>80% of time) | More than half  the time  (50-80% of time) | Less than half  the time  (<50% of time) | Procured externally by patient/family | Never |
| --- | --- | --- | --- | --- | --- |
| Labetolol | 🞏 | 🞏 | 🞏 | 🞏 | 🞏 |
| Hydralazine | 🞏 | 🞏 | 🞏 | 🞏 | 🞏 |
| Nifedipine | 🞏 | 🞏 | 🞏 | 🞏 | 🞏 |
| Methyldopa | 🞏 | 🞏 | 🞏 | 🞏 | 🞏 |

Please list any additional comments here:

55. Is **magnesium** sulfate free of charge to patients at facilities that offer maternity services?

|  | Yes | No |
| --- | --- | --- |
| Public facilities | 🞏 | 🞏 |
| Private facilities | 🞏 | 🞏 |

56. How frequently do stock-outs of **magnesium sulfate** occur?

|  | Frequently (once in every  2 months) | Sometimes (every 3-6 months) | Rarely  (once a year) |
| --- | --- | --- | --- |
| At the central level | 🞏 | 🞏 | 🞏 |
| At the district/regional level | 🞏 | 🞏 | 🞏 |

57. Is there a system in place to ensure a **50% solution of magnesium sulfate** is available in country?

|  | Yes | No |
| --- | --- | --- |
| Public facilities | 🞏 | 🞏 |
| Private facilities | 🞏 | 🞏 |

58. Is there a logistics management system independent of the national procurement system that private sector facilities use to procure essential medications?

🞏 Yes	🞏 No

59. From where does the private sector source HDP commodities? Select all the apply:

🞏 Received free from the government central medical store

🞏 Government deliveries

🞏 Private sector goes to source to collect

🞏 Other (please specify):

60. How does the private sector receive and distribute HDP commodities? Select all that apply:

🞏 Received free from donors

🞏 Received free from donors

🞏 Private sector has wholesaler/ other distributor delivery

🞏 Other (please specify):

### NATIONAL REPORTING OF MH INDICATOR

61. Is an indicator for the number of women with severe pre-eclampsia/eclampsia included in the national Health Management Information Systems (HMIS)?

🞏 Yes	🞏 No

62. Does the private sector report on number of women with **severe pre-eclampsia/ eclampsia** on the national HMIS?

🞏 Yes	🞏 No

If no, why not?:

63. Are there national level accountability mechanisms that link reporting of the national HMIS indicator to implementation of the policy and guidelines for HDP prevention and treatment at the facility level?

🞏 Yes	🞏 No

If yes, please describe:

### PROGRAMMING

64. Which activities in HDP prevention and management are being supported by the Ministry of Health (MOH), World Health Organization (WHO), UNICEF, UNFPA, and/or other multilateral implementing partners? Select all that apply:

🞏 Health system governance (health service financing, eliminating policy barriers)

🞏 National policy (essential medicine list, national policies and service delivery guidelines consistent with current WHO standards)

🞏 Drugs and equipment (logistics, procurement, quality assurance)

🞏 Service delivery capacity at sites (reliable infrastructure, personnel and systems to deliver services)

🞏 Health worker pre-service and in-service training (competency based, curricula consistent with WHO)

🞏 Partnership development (private/public sector, professional associations, local government, non-governmental organizations, MOH collaboration)

🞏 Community awareness of maternal newborn health issues (civil society, women’s group advocacy for early recognition of problems and referral)

🞏 Health technology and information systems (improved devices, procedures, systems of data management)

🞏 Other (please specify):

65. Which activities in HDP prevention and management programming are being undertaken by the private sector? Select all that apply

🞏 Health system governance (health service financing, eliminating policy barriers)

🞏 National policy (essential medicine list, national policies and service delivery guidelines consistent with current WHO standards)

🞏 Drugs and equipment (logistics, procurement, quality assurance)

🞏 Service delivery capacity at sites (reliable infrastructure, personnel and systems to deliver services)

🞏 Health worker pre-service and in-service training (competency based, curricula consistent with WHO)

🞏 Partnership development (private/public sector, professional associations, local government, non-governmental organizations, MOH collaboration)

🞏 Community awareness of maternal newborn health issues (civil society, women’s group advocacy for early recognition of problems and referral)

🞏 Health technology and information systems (improved devices, procedures, systems of data management)

🞏 Other (please specify):

66. What percent of districts are covered by current HDP programs? (Provide your best possible estimate)

🞏 90-100%

🞏 80-90%

🞏 70-80%

🞏 60-70%

🞏 50-60%

🞏 Less than 50%

### OPPORTUNITIES FOR EXPANSION AND SCALE-UP

67. Please describe any potential opportunities that you see for program introduction, expansion or scale up in the public and private sectors.

68. What opportunities are there for the public and private sector to collaborate on HDP? Select all that apply:

🞏 District work planning

🞏 Regular data review

🞏 Maternal death audits

🞏 Civil society initiatives to reduce HDP

🞏 Other (please specify):

69. What are the three most significant bottlenecks to scaling up HDP management programs in your country? Briefly describe what is being done or could be done to address the bottlenecks.

### DEMOGRAPHICS

70. Which country do you represent?

71. What was the approximate number of respondents to the survey?

72. What groups were represented by your key informants? Select all that apply: Ministry of health

🞏 Obstetrics-gynaecology professional association

🞏 Nursing professional association

🞏 Midwifery professional association

🞏 Obstetrics-gynaecology education council

🞏 Nursing education council

🞏 Midwifery education council

🞏 Non-governmental organizations

🞏 Faith-based organizations

🞏 For-profit organizations

🞏 Other (please specify):

73. In responding to questions in this survey about the private sector, how did you determine your response? Select all that apply:

🞏 Key private sector stakeholders were involved in consultation

🞏 Associated with data captured in a national database

🞏 An educated estimate

🞏 No available data

🞏 Other (please specify):

74. Is there any additional information you would like us to know?
