# Supplementary material for: Prevention and management of postpartum hemorrhage and hypertensive disorders of pregnancy: results from a 31-country rapid assessment
Source: BMC Pregnancy Childbirth. 2026 Jul 20;26:1191. doi: 10.1186/s12884-026-09679-w (PMC13681778; doi:10.1186/s12884-026-09679-w)
Supplement: Supplementary file 2 [file 12884_2026_9679_MOESM2_ESM.docx]

Additional File 2: Composite scores by theme

*Note: for each theme (column), a “yes” answer on each indicator contributed 1 point. Total points per theme were tallied for each country and divided by the total number of indicators for that theme, then assessed against the 80% composite score threshold.*

| **Composite scoring criteria by theme** | | | | | | | | |
| --- | --- | --- | --- | --- | --- | --- | --- | --- |
| PPH drug availability | HPD drug availability | National guidelines for PPH updated to global management principles | National guidelines for HDP updated to global management principles | Quality and procurement policies at the national level | Midwifery scope of practice | Pre-service capacity building and training in global best practices | In-service capacity building and training in global best practices | National reporting on select MNH indicators |
| Oxytocin on the EML | Labetalol is on the EML for severe PE/E | National guidelines include TXA for treatment of PPH | National guidelines include beta-blocker for non-severe hypertension | System to ensure 50% solution of MgSO4 in the public sector | Public sector midwifery scope includes diagnosing severe pre-eclampsia/ eclampsia and giving initial loading dose of MgSO4 | Public sector training institutions curricula include anti-convulsant management of severe pre-eclampsia/ eclampsia with MgSO4 as first line treatment | Public sector training institutions curricula include anti-convulsant management of severe pre-eclampsia/ eclampsia with MgSO4 as first line treatment | There is national level accountability mechanisms linking reporting of a HDP indicator in the HMIS to policies at facilities J4 |
| Misoprostol on the EML | Hydralazine is on the EML for severe PE/E | National guidelines include UBT for treatment of PPH | National guidelines include hydralazine for non-severe hypertension | System to ensure 50% solution of MgSO4 in the private sector | Private sector midwifery scope includes diagnosing severe pre-eclampsia/ eclampsia and giving initial loading dose of MgSO4 | Private sector training institutions curricula include anti-convulsant management of severe pre-eclampsia/ eclampsia with MgSO4 as first line treatment | Private sector training institutions curricula include anti-convulsant management of severe pre-eclampsia/ eclampsia with MgSO4 as first line treatment | There is national level accountability mechanisms linking reporting of a PPH indicator in the HMIS to policies at facilities |
| Oxytocin stock-outs are rare at central level | Methyldopa is on the EML for severe PE/E | National guidelines include NASG for treatment of PPH | National guidelines include methyldopa for non-severe hypertension | Logistics management system exists for PPH in the private sector | Public sector midwifery scope includes performing manual removal of placenta | Public sector training institutions curricula include calcium supplementation for pregnant women in areas of low dietary calcium for the prevention of pre-eclampsia/ eclampsia | Public sector training institutions curricula include calcium supplementation for pregnant women in areas of low dietary calcium for the prevention of pre-eclampsia/ eclampsia | Indicator exists for number of women with severe pre-eclampsia/ eclampsia in the HMIS |
| Oxytocin stock-outs are rare at district/regional level | Nifedipine is on the EML for severe PE/E | Private sector uses PPH national guidelines | National guidelines include nifedipine for non-severe hypertension | Logistics management system exists for HDP in the private sector | Private sector midwifery scope includes performing manual removal of placenta | Private sector training institutions’ curricula include calcium supplementation for pregnant women in areas of low dietary calcium for the prevention of pre-eclampsia/ eclampsia | Private sector training institutions’ curricula include calcium supplementation for pregnant women in areas of low dietary calcium for the prevention of pre-eclampsia/ eclampsia | Indicator exists for giving uterotonic immediately after delivery in the HMIS |
| Misoprostol is regularly available at public facilities | MgSO4 is on the EML for severe PE/E | National guidelines include oxytocin as preferred uterotonic | National guidelines include hydralazine as first line anti-hypertensive in severe pre-eclampsia/ eclampsia | Public facilities have controlled cold-chain for oxytocin | Public sector midwifery scope includes applying/ removing the non-pneumatic anti-shock garment | Public sector training institutions’ curricula include management of non-severe hypertension of pregnancy with methyldopa or beta blocker | Public sector training institutions’ curricula include management of non-severe hypertension of pregnancy with methyldopa or beta blocker | Private sector reports on the number of women with severe pre-eclampsia/ eclampsia in the HMIS |
| Misoprostol is regularly available at private facilities | MgSO4 is free at public facilities | National policy includes giving misoprostol to women delivering at home or without a skilled birth attendant for PPH prevention | National guidelines include labetalol as first line anti-hypertensive in severe pre-eclampsia/ eclampsia | Private facilities have controlled cold-chain for oxytocin | Private sector midwifery scope includes applying/ removing the non-pneumatic anti-shock garment | Private sector training institutions curricula include management of non-severe hypertension of pregnancy with methyldopa or beta blocker | Private sector training institutions curricula include management of non-severe hypertension of pregnancy with methyldopa or beta blocker | Private sector reports on uterotonic use immediately after delivery on the HMIS |
| Oxytocin is regularly available at public facilities | MgSO4 is free at private facilities | National policy includes updated AMTSL | National guidelines include methyldopa as first line anti-hypertensive in severe pre-eclampsia/ eclampsia | National procurement policy exists for oxytocin | Public sector midwifery scope includes giving tranexamic acid | Public sector training institutions curricula include management of severe hypertension of pregnancy with labetalol, hydralazine, nifedipine, or methyldopa | Public sector training institutions curricula include management of severe hypertension of pregnancy with labetalol, hydralazine, nifedipine, or methyldopa |  |
| Oxytocin is regularly available at private facilities | MgSO4 is regularly available at public facilities | National policy for blood transfusion services exists | National guidelines include nifedipine as first line anti-hypertensive in severe pre-eclampsia/ eclampsia | National procurement policy exists for misoprostol | Private sector midwife scope includes giving tranexamic acid | Private sector training institutions curricula include management of severe hypertension of pregnancy with labetalol, hydralazine, nifedipine, or methyldopa | Private sector training institutions curricula include management of severe hypertension of pregnancy with labetalol, hydralazine, nifedipine, or methyldopa |  |
| Oxytocin is free at public facilities | MgSO4 is regularly available at private facilities | National guidelines includes ergometrine/ methylergometrine if oxytocin quality not guaranteed | National guidelines include recommendation on induction of labor for severe pre-eclampsia/ eclampsia before term | National procurement policy exists for ergometrine | Public sector midwife scope includes management of uterine balloon tamponade | Public sector training institutions curricula include oxytocin as preferred uterotonic if quality can be assured, otherwise other first line uterotonic recommended | Public sector training institutions curricula include oxytocin as preferred uterotonic if quality can be assured, otherwise other first line uterotonic recommended |  |
| Oxytocin is free at private facilities | Stock-outs of MgSO4 are rare at central level | National guidelines include oxytocin-ergometrine if oxytocin quality not guaranteed | Private sector uses national HDP guidelines | Distribution, storage, transport policy exists that meets international norms for oxytocin | Private sector midwife scope includes management of uterine balloon tamponade | Private sector training institutions curricula include oxytocin as preferred uterotonic if quality can be assured, otherwise other first line uterotonic recommended | Private sector training institutions curricula include oxytocin as preferred uterotonic if quality can be assured, otherwise other first line uterotonic recommended |  |
|  | Stock-outs of MgSO4 are rare at district/regional level | National guidelines include misoprostol if oxytocin quality not guaranteed | National guidelines include calcium supplementation in pregnancy for women living in areas of low dietary calcium | Distribution, storage, transport policy exists that meets international norms for misoprostol |  | Public sector training institutions curricula include tranexamic acid as treatment for PPH | Public sector training institutions curricula include tranexamic acid as treatment for PPH |  |
|  | Labetalol is regularly available at public facilities |  | National guidelines include management of women with HDP after giving birth | Distribution, storage, transport policy exists that meets international norms for ergometrine |  | Private sector training institutions curricula include tranexamic acid as treatment for PPH | Private sector training institutions curricula include tranexamic acid as treatment for PPH |  |
|  | Labetalol is regularly available at private facilities |  | National guidelines include low dose aspirin for the prevention of pre-eclampsia in high-risk women |  |  | Public sector training institutions curricula include updated Active Management of Third Stage of Labor (AMTSL) | Public sector training institutions curricula include updated AMTSL |  |
|  | Hydralazine is regularly available at public facilities |  | National guidelines include MgSO4 is first line anti-convulsant in severe pre-eclampsia/ eclampsia |  |  | Private sector training institutions curricula include updated AMTSL | Private sector training institutions curricula include updated AMTSL |  |
|  | Hydralazine is regularly available at private facilities |  |  |  |  | Public sector training institutions curricula include uterine balloon tamponade as second line treatment for PPH due to atony in setting with appropriate context | Public sector training institutions curricula include uterine balloon tamponade as second line treatment for PPH due to atony in setting with appropriate context |  |
|  | Methyldopa is regularly available at public facilities+C20 |  |  |  |  | Private sector training institutions curricula include uterine balloon tamponade as second line treatment for PPH due to atony in setting with appropriate context | Private sector training institutions curricula include uterine balloon tamponade as second line treatment for PPH due to atony in setting with appropriate context |  |
|  | Methyldopa is regularly available at private facilities |  |  |  |  | Public sector training institutions curricula include non-pneumatic antishock garment for second line treatment of PPH | Public sector training institutions curricula include non-pneumatic antishock garment for second line treatment of PPH |  |
|  | Nifedipine is regularly available at public facilities |  |  |  |  | Private sector training institutions curricula include non-pneumatic antishock garment for second line treatment of PPH | Private sector training institutions curricula include non-pneumatic antishock garment for second line treatment of PPH |  |
|  | Nifedipine is regularly available at private facilities |  |  |  |  | Public sector training institutions curricula include use of daily low dose aspirin for the prevention of pre-eclampsia in high-risk pregnant women | Public sector training institutions curricula include use of daily low dose aspirin for the prevention of pre-eclampsia in high-risk pregnant women |  |
|  |  |  |  |  |  | Private sector training institutions curricula include use of daily low dose aspirin for the prevention of pre-eclampsia in high-risk pregnant women | Private sector training institutions curricula include use of daily low dose aspirin for the prevention of pre-eclampsia in high-risk pregnant women |  |
